# RNA-sequencing reveals the complexities of the transcriptional response to lignocellulosic biofuel substrates in *Aspergillus niger*

**DOI:** 10.1186/s40694-014-0003-x

**Published:** 2014-11-17

**Authors:** Steven T Pullan, Paul Daly, Stéphane Delmas, Roger Ibbett, Matthew Kokolski, Almar Neiteler, Jolanda M van Munster, Raymond Wilson, Martin J Blythe, Sanyasi Gaddipati, Gregory A Tucker, David B Archer

**Affiliations:** 1 School of Life Sciences, University of Nottingham, University Park, Nottingham, NG7 2RD UK; 2 School of Biosciences, University of Nottingham, Sutton Bonington Campus, Loughborough, LE12 5RD UK; 3 Deep Seq, Faculty of Medicine and Health Sciences, Queen’s Medical Centre, University of Nottingham, Nottingham, NG7 2UH UK; 4 Present Address: Microbiology Services, Public Health England, Salisbury, UK; 5 Present Address: Sorbonne Universités, UPMC Univ Paris 06, IBPS UMR 7138, Evolution Paris-Seine, Paris, F-75005 France

**Keywords:** Biofuels, RNA-seq, Aspergillus, Wheat straw, Willow, Transcriptome

## Abstract

**Background:**

Saprobic fungi are the predominant industrial sources of Carbohydrate Active enZymes (CAZymes) used for the saccharification of lignocellulose during the production of second generation biofuels. The production of more effective enzyme cocktails is a key objective for efficient biofuel production. To achieve this objective, it is crucial to understand the response of fungi to lignocellulose substrates. Our previous study used RNA-seq to identify the genes induced in *Aspergillus niger* in response to wheat straw, a biofuel feedstock, and showed that the range of genes induced was greater than previously seen with simple inducers.

**Results:**

In this work we used RNA-seq to identify the genes induced in *A. niger* in response to short rotation coppice willow and compared this with the response to wheat straw from our previous study, at the same time-point. The response to willow showed a large increase in expression of genes encoding CAZymes. Genes encoding the major activities required to saccharify lignocellulose were induced on willow such as endoglucanases, cellobiohydrolases and xylanases. The transcriptome response to willow had many similarities with the response to straw with some significant differences in the expression levels of individual genes which are discussed in relation to differences in substrate composition or other factors. Differences in transcript levels include higher levels on wheat straw from genes encoding enzymes classified as members of GH62 (an arabinofuranosidase) and CE1 (a feruloyl esterase) CAZy families whereas two genes encoding endoglucanases classified as members of the GH5 family had higher transcript levels when exposed to willow. There were changes in the cocktail of enzymes secreted by *A. niger* when cultured with willow or straw. Assays for particular enzymes as well as saccharification assays were used to compare the enzyme activities of the cocktails. Wheat straw induced an enzyme cocktail that saccharified wheat straw to a greater extent than willow. Genes not encoding CAZymes were also induced on willow such as hydrophobins as well as genes of unknown function. Several genes were identified as promising targets for future study.

**Conclusions:**

By comparing this first study of the global transcriptional response of a fungus to willow with the response to straw, we have shown that the inducing lignocellulosic substrate has a marked effect upon the range of transcripts and enzymes expressed by *A. niger*. The use by industry of complex substrates such as wheat straw or willow could benefit efficient biofuel production.

**Electronic supplementary material:**

The online version of this article (doi:10.1186/s40694-014-0003-x) contains supplementary material, which is available to authorized users.

## Background

The production of second generation biofuels uses lignocellulose as the source of sugars for fermentation [1]. A key problem with using lignocellulose is its recalcitrance to saccharification. This recalcitrance leads to a requirement for large amounts of polysaccharide-degrading enzymes which are costly to produce at the industrial scale. Furthermore, the enzyme cocktails produced mainly by saprobic fungi are usually deficient in activities required for complete saccharification of a particular substrate. One cause of this may be the industrial use of simple, cost effective inducers, such as lactose or cellulose which fail to induce the full range of hydrolytic and accessory activities encoded within an organism’s genome. Differences in the enzyme cocktails expressed in response to complex, lignocellulosic substrates as compared to defined substrates such as pure cellulose have been observed in several fungal species [2]-[5]. Simple inducers may not provide the most effective possible enzyme profile that is achievable from the arsenal encoded within a fungal genome for any given substrate. The effectiveness of the cocktail is also likely to vary towards different substrates, even if the induction mechanism remains elusive. The pairing of inducer and lignocellulosic substrate is therefore a key consideration in biofuel production.

The perspective of how plants and fungi evolved provides a context for improvement of the efficiency of saccharification. Lignocellulose is present in plant secondary cell walls and is mainly composed of cellulose, hemicelluloses and lignin [1]. Generally, plants that are evolutionarily close share a more similar lignocellulose composition and this is particularly evident in hemicellulose composition [6]. At the same time, fungi have evolved complex regulatory mechanisms to sense their environment including nutrients such as lignocellulose [7]. This is evidenced by the response of fungi to the presence of different polysaccharides such as xylan, arabinan or pectin and suggests that fungi may have specific degradative responses to lignocellulosic substrates from different plant species [8].

We chose to study the response of a fungus to two plant species using *Aspergillus niger* as the fungus and wheat (*Triticum aestivum*) and willow (*Salix* sp.) as the plant species for three key reasons. Firstly, wheat (a grass) and willow (a tree) represent the two major lineages of flowering plants which have evolved cell walls with diverse compositions. Secondly, both species are of potential industrial relevance as feedstocks for biofuel production [9]. Thirdly, *A. niger* is an industrially-relevant model fungus with a large repertoire of CAZymes [10]. Recent studies have highlighted the potential of *A. niger* to respond differently at the transcriptional level to different polysaccharides [5],[11] making it ideally suited to investigate responses to different substrates.

RNA-seq is a method which is transforming how transcriptomes are studied [12] and provides a highly sensitive read-out of responses to substrates at the genome-wide level. Our previous studies using RNA-seq have defined and compared the responses of *A. niger* and *T. reesei* to wheat straw [5],[13],[14]. Another study by Hakkinen et al. [3], in *T. reesei* showed differences and similarities in the transcriptional response to different lignocellulosic substrates. One limitation of that and other studies is the use of microarrays which have a narrower range for which expression can be measured compared to RNA-seq. Also, substrates can be used that have been subjected to harsh pre-treatments, albeit industrially relevant, which alter the substrate far from what fungi have evolved to sense and degrade in nature.

To our knowledge, there is no study that reports the transcriptional response of a fungus to willow, a perennial species that has much potential as a bioenergy crop [9]. The use of the same experimental conditions for culturing *A. niger* on straw and willow presented a unique opportunity to compare the transcriptional responses of *A. niger* to these substrates. These transcriptional responses were compared with biochemical assays of the protein mixes secreted by *A. niger* when cultured with the different substrates.

Here we report on differences and similarities in the transcriptional responses and the secreted protein activities of *A. niger* exposed to wheat straw or willow. We show that the fungal response is to some extent specific to the inducing substrate. Moreover, our results highlight the importance of using complex inducing substrates for production of enzymes and accessory proteins by industry or for discovering the relevant small inducing molecules. Finally, we discuss how biotechnology could be used to benefit biofuels by making better fungal strains.

## Results and discussion

### The willow-induced CAZy expression profile

*A. niger* was cultured in a glucose medium for 48 h followed by transfer to a medium where ball-milled willow was the sole carbon source for 24 h. The sugar composition of the willow substrate (as % of total solids) was 38.5% (±0.90) glucose, 10.4% (±1.03) xylose and 1.1% arabinose (±0.02) and the lignin content was 23.1% (±0.95) (the values in parentheses are standard deviations). To re-introduce a simple carbon source in the presence of lignocellulose, glucose was added to the willow cultures after 24 h and incubated for a further 5 h. RNA was extracted and sequenced from these three conditions. Reads including those mapping to genes encoding enzymes defined by CAZy [15],[16] as being either glycoside hydrolases, polysaccharide lyases, carbohydrate esterases or auxiliary activity proteins, were counted and the relative expression of each family was calculated.

**Figure 1:**
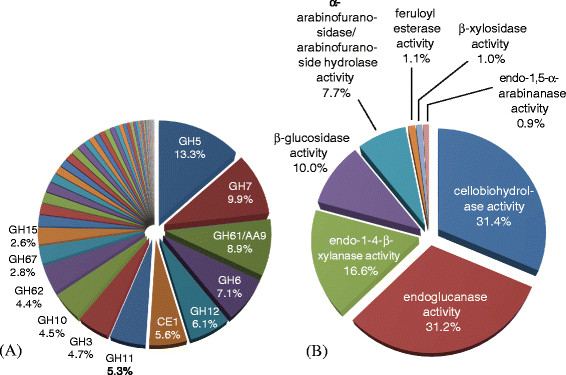
**Percentage of transcripts belonging to (A) CAZy families and (B) particular enzyme activities.** Expression levels were measured following 24 h of culturing *A. niger* in media containing ball-milled willow as the only carbon source. CAZy families representing more than 2% of total CAZy expression are indicated. The percentage values are the RPKM values for **(A)** particular CAZy families expressed as a proportion of the total RPKM value of all the families in the GH, CE, PL and AA classes or **(B)** particular enzyme activities expressed as a proportion of the total RPKM value of all the activities analysed. Classifications for CAZy are according to CAZy.org. Genes were assigned to particular enzyme activities as described in the Methods section and Additional file 7.

Figure 1 shows that after culturing *A. niger* for 24 h with willow, the most abundantly expressed family is GH5, which accounts for 13.3% of total CAZy RPKM (Reads Per Kilobase per Million mapped reads). The GH5 family is divided into a range of subfamilies and encompasses a variety of functions [17]. The majority of GH5 family expression seen in this study originates from two endoglucanase-encoding genes, TID_205580 and TID_209376 (*eglB*), which are expressed at 121 and 210 RPKM respectively. The expression values of the genes described in this section are listed in Table 1 and a full list of individual gene expression values can be found in the Additional file 1.

**Table 1:** **Expression of key CAZy-encoding genes that are induced on the willow substrate**

Gene ID		Annotation ^a^	CAZy family		RPKM	
ATCC 1015	CBS513.88			Glucose 48 h	Willow ^b^ 24 h	Willow 24 h + Glucose 5 h
TID_211595	An12g04610	Lytic polysaccharide monooxygenase	AA9	0.6	246.9	1.2
TID_209376	An07g08950	*eglB* - Heat- and alkaline-stable endoglucanase	GH5	0.5	210.1	0.7
TID_211544	An12g05010	*axeA* - Acetyl xylan esterase	CE1	0.6	161.5	0.3
TID_53159	An07g09330	*cbhA* - Putative cellobiohydrolase	GH7	1.0	155.7	1.2
TID_54490	An12g02220	Putative cellulose 1,4-beta-cellobiosidase	CBM1, GH6	0.3	145.2	0.4
TID_205580	An01g11670	Endoglucanase A	GH5, CBM1	0.2	120.6	1.6
TID_211053	An14g02760	*eglA* - Putative secreted endoglucanase A	GH12	0.4	116.3	1.4
TID_51773	An01g11660	*cbhB* - Putative cellobiohydrolase B	GH7, CBM1	0.3	115.9	10.8
TID_133986	An08g01760	Putative cellulase; exocellobiohydrolase	GH6	0.2	54.9	0.5
TID_52011	An01g03340	Xyloglucan-specific endo-beta-1,4-glucanase	GH12	0.5	49.3	0.7

^a^The annotations here are for the *A. niger* CBS513.88 strain from the AspGD database [44] with the exception that the erroneous endoglucanase annotation for the AA9 family member was corrected.

^b^All of the genes listed here have significantly increased expression on the willow substrate compared to the glucose 48 h control (*p* < 0.05, DESeq).

The two GH7, reducing-end-active, cellobiohydrolases encoded within the *A. niger* genome, CbhA and CbhB (the products of TID_53159 and TID_51773), are expressed at 156 and 116 RPKM respectively, and together represent 9.9% of CAZy gene expression. The non-reducing end processive cellobiohydrolases of the GH6 family are also expressed highly due to expression of TID_54490 at 145 RPKM and TID_133986 at 55 RPKM.

Expression of two genes encoding GH12 family enzymes, TID_211053, encoding the endoglucanase EglA, and TID_52011 encoding an enzyme with strong similarity to xyloglucan-specific endo-beta-1,4-glucanase from *Aspergillus aculeatus* [18],[19], at 116 and 49 RPKM respectively account for 6.1% of CAZy expression.

In addition to the glycoside hydrolase families, the AA9 (formerly GH61 [20]) family of lytic polysaccharide monooxygenases, is also highly expressed and accounts for 8.9% of CAZy expression. This is almost completely due to the expression of TID_211595 at 247 RPKM. A 5.6% contribution is also made by the CE1 family of carbohydrate esterases due to high levels of transcription of a single gene, the acetyl xylan esterase, *axeA* (TID_211544) at 161 RPKM.

This transcript distribution broadly reflects the enzymes required for the saccharification of lignocellulose. The key enzymes involved in the bulk degradation of the cellulose and xylan backbones, i.e. endoglucanases and cellobiohydrolases in the former case and xylanases in the latter, make up the greatest proportion of CAZy expression. Expressed at a lower, but still substantial level, are accessory proteins such as the AA9 family lytic polysaccharide monooxygenases, that are thought to improve the accessibility to the recalcitrant polymers and play a role in depolymerisation of cellulose through oxidative cleavage [21],[22]. CE1 esterases, which cleave the acetyl group linkages of xylans [23] are also expressed at a lower, but still sizable level. Notably, there is relatively little expression of the β-glucosidases and xylosidases when *A. niger* is exposed to willow.

### A cross-substrate comparison using transcriptomics

In an earlier study [5] we delineated the transcriptional profile of *A. niger* under conditions identical to those used here, with the exception that wheat straw was provided as the only lignocellulosic substrate. The composition of the wheat straw (as % of total solids and using the same compositional analysis methods as used for willow) was 37.4% (±2.96) glucose, 16.8% xylose (±2.48) and 2.7% arabinose (±0.29) and the lignin content was 21.6% (±0.73) (the values in parentheses are standard deviations). Re-calculation of the expression data using the updated genome model and method outlined in this manuscript, allows the direct comparison of the two datasets. A time-dependent transcriptome profile on the two different substrates was not feasible but we accept that the transcript profiles may change with time differently on wheat straw or willow. Therefore, we interpret the comparative data cautiously and focus on the major differences for discussion.

**Figure 2:**
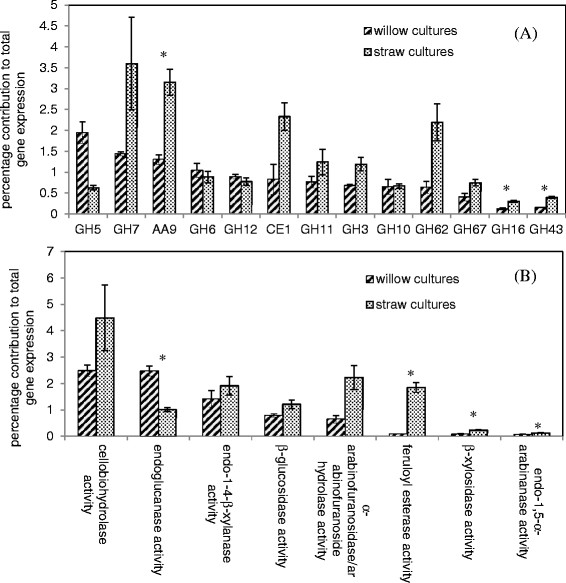
**Expression per (A) CAZy family and (B) selected enzyme activities as a percentage of total gene expression.** For **(A)**, only those families with total RPKM values of 100 or more upon at least one of the substrates are shown. Gene expression was measured following 24 h culturing of *A. niger* in the presence of willow or wheat straw as the only carbon source. The families and activities are presented in order of decreasing expression from the willow cultures. The error bars represent standard error. Genes were assigned to particular enzyme activities as described in Methods section and Additional file 7. A ‘*’ symbol is used to indicate whether the differences between willow and straw cultures were statistically significant (unequal variances *t*-test, *p* <0.05).

Following 24 hours exposure to each substrate, the total CAZy expression induced by willow contributes 15% of total gene expression, while the equivalent for wheat straw is 21%. Therefore, the CAZy portion of the transcriptome is approximately one third greater in response to wheat straw than willow at this specific time point. Figure 2 compares the relative contribution to total gene expression under each condition per CAZy family (for those families in which a total of at least 100 RPKM was achieved on either substrate) and also per selected enzyme activities that the genes encode. There are similarities in the general expression pattern observed, with GH6, 7, 11, 12, CE1 and AA9 being amongst the most highly expressed families on either substrate. For almost all families, relative expression is greater in response to wheat straw than willow, as might be expected when the total expression of CAZy genes overall is one third greater on wheat straw. However, the size of the increase in expression of the CAZy families is not uniform, implying that the difference between the two responses is not simply correlated with the total CAZy expression but instead due to differences in the levels of induction of genes in the CAZy families. In the CE1 family, for example, a single acetylxylan esterase gene, *axeA*, is highly expressed following exposure to willow (161 RPKM). The gene *axeA* is also highly expressed following exposure to straw (562 RPKM) with no statistically significant difference in expression compared to willow (DESeq, P = 0.89). However a second CE1 family member, TID_43785 which is the ortholog of the feruloyl esterase *faeC* from *Aspergillus nidulans* [24], is expressed poorly on willow (3 RPKM) but is highly induced (325 RPKM) on wheat straw with a significant 53-fold (DESeq, *p* < 0.01) increase in expression on straw compared to willow (Table 2 and Additional file 1).

**Table 2:** **Expression of genes that are relevant to the straw and willow substrate comparison**

Gene ID	Annotation ^a^	RPKM				Fold change ^b^ straw/willow	***p-*** value straw/willow
ATCC 1015		Glucose 48 h	Straw 24 h	Glucose 48 h	Willow 24 h		
TID_43785	Feruloyl esterase	0.4	325.9	0.0	3.1	52.6	<0.01
TID_182100	Has domain(s) with predicted hydrolase activity	0.1	16.8	0.0	0.3	28.4	<0.01
TID_51662	*faeA* - Feruloyl esterase	0.4	396.3	0.0	13.0	13.9	<0.01
TID_47677	Putative xylan beta-xylosidase	0.2	76.0	0.1	9.4	3.7	<0.01
TID_55136	*axhA*, 1,4-beta-D-arabinoxylan arabinofuranohydrolase	1.7	904.6	0.9	116.3	3.4	0.15
TID_211544	*axeA*, Acetyl xylan esterase	0.7	562.4	0.6	161.5	1.7	0.83
TID_51478	*faeB* - Feruloyl esterase	0.3	1.9	0.5	0.5	1.6	0.64
TID_205580	Endoglucanase A	0.1	73.7	0.2	120.6	0.3	<0.05
TID_209376	Heat- and alkaline-stable endoglucanase	0.1	113.6	0.5	210.1	0.3	<0.01

^a^The annotations here are for the *A. niger* CBS513.88 strain from the AspGD database [44].

^b^The fold changes were calculated by the DESeq statistical analysis package using the normalised read counts.

The two most striking differences seen in the pattern of expression of genes encoding entire CAZy enzyme families are in GH62 and GH5. GH62 family members are arabinofuranosidases, a single example of which is encoded within the *A. niger* genome, the 1,4-β-D-arabinoxylan arabinofuranohydrolase, *axhA* (TID_55136) [25], which is expressed at 116 RPKM on willow. The GH62 family is the tenth most expressed family on willow whereas in contrast it is the third most expressed family on straw. This difference in the ranking order of the GH62 family is due to a 3.4 fold increase in expression of *axhA* (900 RPKM) on straw compared to willow. This trend in expression with the GH62 family member *axhA* has been confirmed by qRT-PCR (Additional file 2).

The GH5 family is notable amongst the most highly expressed families for being the only one in which greater expression is seen in response to willow. Whilst the two predominant endoglucanases expressed on willow, TID_205580 and TID_209376 (*eglC*), remain the two most highly expressed members of the family on wheat straw, levels are ~3-fold reduced for each as calculated using DESeq1.9.

The overall responses to these two different substrates, represented by the hardwood and straw feedstocks, retain a core set of highly induced genes that encode enzymes required for the degradation of any lignocellulosic substrate. Regulatory differences at the level of individual genes could have led to the substrate-specific variations in gene expression, which fine-tuned the response of the cells for the degradation of the particular substrate. These variations in gene expression are possibly due to differences in the inducing signals that are generated from the substrates, probably through the generation of small inducing molecules (e.g. sugars and sugar oligomers) from the respective feedstocks. Wheat (a graminaceous (grass) monocot) and willow (a woody dicot) lineages are separated by ~400 million years of evolution and in that time have evolved different plant cell wall properties.

At the molecular level one of the notable differences between the hemicelluloses of grasses and dicots (reviewed in [6]) is the increased decoration of glucuronoxylan in grasses with arabinose to form glucuronoarabinoxylan. The increased levels of arabinoxylans within grasses may explain the particularly increased expression level seen for the GH62 family on wheat straw as compared to willow. The enzymes within this family are arabinofuranosidases that act to cleave arabinofuranose side chains from the xylan backbone of arabinoxylans [25]. GH43 also includes arabinofuranosidases, many of which display both α-L-arabinosidase and β-D-xylosidase activity. Although the overall expression level of this family in these experiments was relatively low on both substrates, there was significantly increased expression on wheat straw compared to willow of two genes, TID_182100 and TID_47677, which were both significantly more highly expressed (28-fold and 4-fold increased respectively, analysed using DESeq1.9, *p* < 0.001). Arabinose-responsive transcriptional regulation of CAZy enzymes through the AraR regulatory pathway has been described previously in *A. niger* [26],[27].

In addition to hemicellulose composition, the cross-linking between cell wall components also differs between wheat and willow; in grasses such as wheat, ferulic acid linkages connect lignin and hemicelluloses but are largely absent from dicots such as willow [28],[29]. There are three genes encoding enzymes with potential ferulic acid esterase activity within the *A. niger* genome *faeA*, *faeB* and TID_43785. The gene *faeA* (TID_51662) has been demonstrated by de Vries and colleagues [30] to encode a ferulate esterase, although it is not officially classified as a carbohydrate esterase by CAZy. After exposure to wheat straw [5], *faeA* was one of the most highly expressed genes and the expression level as calculated for this study is 396 RPKM. In comparison, the expression of *faeA* on willow is 13.9-fold lower than the expression on straw. The expression levels of *faeA* were demonstrated to be regulated by both xylose/xylan and ferulic acid specific systems, with induction stimulated by xylose alone but optimal induction occurring in the presence of ferulic acid in addition to xylose [31]. FaeB (encoded by TID_51478), which is not expressed over 2 RPKM on either substrate has significantly decreased activity towards wheat straw arabinoxylan compared to FaeA [32]. As discussed previously, the putative feruloyl esterase encoded by TID_43785 is one of the most highly differentially expressed genes between the two substrates. The increased expression on wheat straw could indicate that it shares a ferulate-dependent induction system with *faeA*. The same trend was observed for TID_43785 and *faeA* using qRT-PCR with a repeat experiment replicating the conditions used for RNA-seq (Additional file 2). These transcriptional observations support the hypothesis that the response of *A. niger* to lignocellulose is fine-tuned by, and linked to, the substrate composition.

### Comparison of culture supernatant activity using azurine dyed substrates

The RNA-seq data suggested transcriptional specificity in the response of *A. niger* to each substrate. To investigate this biochemically, supernatants harvested from *A. niger* cultures incubated with either substrate for 24 h (the same conditions used for RNA-seq) were assayed for activity towards two azurine-linked substrates: cellulose – a major component of both the wheat straw and willow substrates and arabinoxylan – a hemicellulose found in much greater abundance in wheat straw than willow. Activities in the culture supernatants were assayed using a predetermined volume of the supernatants that gave a linear response between time and azurine dye release. The activity towards arabinoxylan was 10-fold greater in the straw-induced cocktail compared to the willow-induced cocktail (Table 3). To demonstrate that this higher level of activity towards arabinoxylan could not just be explained by higher total amounts of lignocellulosic active enzymes in the straw cocktail, the activity towards arabinoxylan was expressed as a ratio of the activity towards HE-cellulose. This ratio showed that there was still a greater level of arabinoxylan activity in the straw-induced cocktail because the ratio of the activities was 4-fold greater in the straw-induced cocktail compared to the willow-induced cocktail (Table 3). The greater activity towards arabinoxylan in the wheat straw-induced cocktail has support from the RNA-seq data where the GH62 family gene *axhA* encoding arabinofuranosidase activity has higher expression in *A. niger* wheat straw-induced cultures compared to the willow-induced cultures. For further investigation of enzyme activities, the supernatants were concentrated so as to allow estimation of the amount of protein and they were then used to saccharify either wheat straw or willow.

**Table 3:** **Activity towards AZCL HE-cellulose and AZCL arabinoxylan from culture supernatants (S/Ns)**

	HE-cellulose ^a^	AZCL-arabinoxylan ^a^	ratio AZCL-arabinoxylan/HE-cellulose
S/N from *A. niger* cultured with straw	0.031 (0.0015)^b^	1.046 (0.0228)^b^	**34.05**
S/N from *A. niger* cultured with willow	0.010 (0.0002)^b^	0.082 (0.0002)^b^	**8.17**

^a^activity is expressed as absorbance units/μl of culture S/N.

^b^values in parentheses are standard errors.

### Wheat straw induces a greater and different secretory response than willow

The protein concentration estimated within the concentrated supernatant from wheat straw cultures was approximately twice that from equivalent willow cultures. SDS-PAGE of the two sets of supernatants demonstrated highly reproducible protein mixtures across replicate samples. Whilst some major bands were conserved, clear differences were observed in the banding patterns between the two sets of conditions reflecting the general pattern seen in the transcriptomic data (Additional file 3).

### Comparison of culture supernatant activities using *para*-nitrophenyl linked substrates

**Figure 3:**
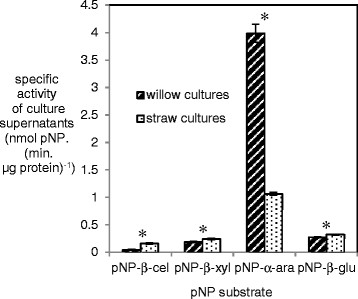
**Specific activity towards** ***para*** **-nitrophenyl (pNP) substrates from supernatants from** ***A. niger*** **cultured with either wheat straw or willow.** A ‘*’ symbol is used to indicate whether the differences between willow and straw cultures were statistically significant (Student’s *t*-test*, p* < 0.05). Abbreviations are: pNP-β-cel: 4-Nitrophenyl-β-D-cellobioside; pNP-α-ara: 4-Nitrophenyl-α-L-arabinofuranoside; pNP-β-glu; 4-Nitrophenyl-β-D-glucopyranoside and pNP-β-xyl: 4-Nitrophenyl-β-D-xylopyranoside. The error bars represent standard errors.

Four *para*-nitrophenyl linked substrates were used to measure specific enzyme activities in the supernatants from the *A. niger* cultures with straw or willow (Figure 3). Slightly higher specific β-glucosidase (1.28 fold) and β-xylosidase (1.17 fold) activities were measured in the straw culture supernatants compared to the willow culture supernatants. The cellobiohydrolase specific activity was also higher (~3.5 fold) in the straw culture supernatants. In contrast, the specific α-arabinofuranosidase/arabinofuranoside hydrolase activity was ~ 2.5 fold lower in the straw culture supernatants compared to the willow culture supernatants. When these specific activities were compared with the RNAseq data from Figure 2, there were similarities in the trends. There was a significantly higher proportion of expression of genes encoding β-xylosidase activity in the straw cultures. Expression of genes encoding for cellobiohydrolase activity or β-glucosidase activity was not significantly different in the straw cultures compared to the willow cultures and neither was there a significant difference in the expression of genes encoding for α-arabinofuranosidase/arabinofuranoside hydrolase activity between the two cultures although that comparison is complicated by the fact that the majority of the gene expression was from *axhA* and AxhA is unable to hydrolyse the pNP-α-arabinofuranoside.

### Comparison of culture supernatant activities using saccharification assays

**Figure 4:**
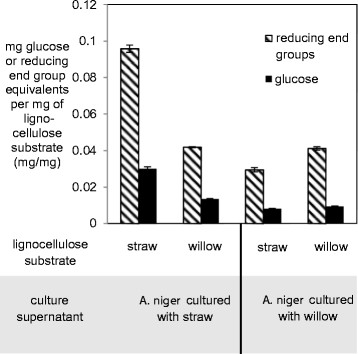
**Saccharification of lignocellulosic substrates by supernatants (S/Ns) from** ***A. niger*** **cultured with either wheat straw or willow.** An equal amount of protein from concentrated S/Ns from *A. niger* cultured with either straw or willow was used to saccharify straw or willow substrates. The glucose and reducing end group equivalents were quantified with the GODPOD and DNS assays respectively. The glucose or reducing end groups released by equal amounts of protein from the different culture S/Ns in a 24 h period are expressed per mg of the lignocellulosic substrate in the saccharification assay. The results are the mean of assays from S/Ns from 3 independent cultures with either lignocellulosic substrate and the error bars represent standard errors.

Over a 48 h saccharification time course using varying concentrations of protein from supernatants of straw or willow cultures, the release of reducing end group equivalents from the straw or willow substrates was periodically assessed using the DNS assay [33] and free glucose levels measured using the GODPOD assay. After 24 h the levels of free sugars measured were over 80% of the total measured after 48 h, and therefore this incubation time was chosen as the standard for all further assays (Additional file 4). Over a 24 h period concentrated samples of supernatant from *A. niger* exposed to wheat straw, containing 55 μg of secreted proteins, released on average 0.096 mg of reducing end group equivalents per mg of wheat straw substrate (as measured by the DNS assay) whilst only 0.042 mg of reducing end group equivalents per mg of willow was released over the same time period (Figure 4). Similarly the supernatant taken from *A. niger* exposed to willow (which contained an equivalent amount of protein to the supernatant assayed from *A. niger* exposed to straw) released 0.042 mg of reducing end group equivalents per mg of willow substrate, but only 0.030 mg was released per mg of wheat straw. These results show that the wheat straw-induced enzyme cocktail had over double the activity towards wheat straw than it does towards willow, and the willow-induced enzyme cocktail shows a 40% greater activity towards willow compared to wheat straw. Both of these differences are statistically significant (*p* < 0.05, Student’s *t*-test). Use of the GODPOD assay for free glucose monomers present within the digest products showed a similar pattern to the DNS assay (Figure 4) at approximately one third of the levels seen in the DNS assay. These proportions may indicate that the primary target of degradative activity in the assays was hemicellulose rather than the glucose-rich cellulosic fraction or that a significant number of oligomers are being released. These assay values are comparable to those in other studies with similar methodologies [34].

### The lignocellulosic substrate used can have a specific effect on induction

The data presented in this study show that the lignocellulosic substrate used to induce the expression of genes encoding degradative enzymes can have a specific effect on the combination of enzymes produced. Wheat straw and willow induced a central core set of shared genes that are likely to be induced by a wide variety of lignocellulosic substrates, but also showed significant differences in the induction of genes encoding both particular GH families and individual genes. This is likely due to the integration of several different regulatory pathways that respond to either different inducing molecules present within the substrates, or else the differences in concentration or proportions of such inducing molecules. Such a system may have evolved to give *A. niger* the ability to adapt its degradative response to match the available substrate within its natural environment. Of the two ball-milled substrates compared in this study, wheat straw led to a larger scale response at the transcriptomic level that was matched by an increased concentration of proteins within the culture supernatant.

With regard to the enzyme activities, the straw-induced enzyme cocktail out-performed the willow-induced cocktail. As well as the wheat straw-induced cocktail saccharifying straw to a greater extent than the willow-induced cocktail, the straw-induced cocktail saccharified willow to the same extent as the willow-induced cocktail. Both cocktails released almost identical amounts of reducing end group equivalents from willow in the DNS assay (Figure 4). The out-performing by the straw-induced cocktail of the willow-induced cocktail may reflect the intrinsically greater recalcitrance of a woody substrate such as willow over a gramineous substrate such as straw rather than differences in specificities of the cocktails. It is possible that any specificity in the willow-induced cocktail towards willow is masked by this greater recalcitrance of the willow substrate.

### Effect of sugars initially present and released over time in the lignocellulose media

The insoluble polymers and soluble inducing molecules related to these polymers may not be the only factors leading to differences in gene expression. There may be a role for the higher amount of free glucose present initially and released in early time points from the willow media compared to the straw media by *A. niger*. This glucose could delay the onset of induction of CAZy genes longer than in the straw media due to CreA repression which would only be alleviated when the free glucose became exhausted. Sugar analysis showed that there was a greater amount of free glucose present initially and released from the substrate by *A. niger* over time in the willow media compared to the straw media (Additional file 5) [5]. Differences in the timing of transcriptional induction and enzyme secretion, as well as with fungal growth rate, could be investigated in future work with straw and willow substrates. This free glucose and other sugars, while a complicating factor in comparing the response to different substrates, are unavoidable because performing some form of extraction or washing of the lignocellulose substrate will likely remove other small soluble molecules relevant to induction. Even so, the level of free glucose in lignocellulosic substrates initially and released over time are variables to consider when comparing the fungal response to different lignocellulosic substrates. In the study reported here, the levels of free glucose at the outset were very low compared to the amount of fungal biomass added and would not support a significant increase in fungal biomass.

### Non-CAZy responses to lignocellulose

In a previous wheat straw study, we identified 28 genes not categorised as members of any CAZy category that had significantly higher transcript levels in response to wheat straw, using a cut-off of a minimum 20-fold induction relative to expression in the glucose media [5]. We divided those genes into four defined categories, encoding: esterases and lipases, surface-interacting proteins, carbon and nitrogen metabolising enzymes, and transporters. The expression of these 28 genes was examined in *A. niger* after exposure to willow for 24 h (Table 4 and Additional file 1). Of the 7 genes included in the lipase/esterase category, four genes were induced significantly by at least 50-fold following the switch from glucose to willow media and were expressed at an RPKM of >10. Some of the encoded proteins have been biochemically characterised as possessing lignocellulosic degrading activity (e.g. the ferulic acid esterase, FaeA [30]). For two uncharacterised genes TID_173684 and TID_54865, our data support the notion that they could function in the degradation of lignocelluloses and are induced by a factor common to wheat straw and willow.

**Table 4:** **Expression of genes relevant to the non-CAZy response of** ***A. niger*** **to willow**

Gene ID			RPKM			Fold change ^b^ Willow 24 h/glucose 48 h	***p-*** value Willow 24 h/glucose 48 h
ATCC 1015	CBS513.88	Gene name (if any) and annotation ^a^ (abbreviated)	Glucose 48 h	Willow 24 h	Willow 24 h + Glucose 5 h		
**Esterases & Lipases**							
TID_173684	An02g09690	Ortholog(s) have role in fatty acid catabolic process	0.50	21.96	0.22	98.05	<0.01
TID_51662	An09g00120	*fae* A, Feruloyl esterase	0.00	13.01	0.06	N/A	<0.01
TID_50877	An13g01880	Triacylglycerol lipase	0.00	0.98	0.00	N/A	<0.01
TID_210730	An16g01880	*lipanl*, Lysophospholipase	0.36	62.07	0.72	379.56	<0.01
TID_54865	N/A	N/A	0.02	27.43	0.16	2713.67	<0.01
This Study	An03g06560	Triacylglycerol lipase	0.00	0.05	0.00	N/A	0.65
TID_53620	An16g03700	Has domain(s) with predicted hydrolase activity	0.05	11.07	14.25	531.54	<0.01
**Surface-interacting Proteins**							
TID_128530	An07g03340	*hyp1*, Hydrophobin	4.21	2.30	4.92	1.09	1.00
This Study	An08g09880	Putative hydrophobin	1.40	6.33	5.62	9.53	<0.01
TID_188224	An09g00840	Putative cell wall galactomannoprotein	1.31	721.16	2.13	1075.30	<0.01
TID_54125	An18g02730	Ortholog of A. nidulans FGSC A4: AN3257	0.12	3.36	0.73	59.77	<0.01
**Enzymes of Carbon and Nitrogen Metabolism**							
TID_51997	An01g03740	D-xylose reductase	0.25	27.73	0.25	242.13	<0.01
TID_52460	An02g13750	Ortholog(s) have extracellular region localization	2.60	35.39	6.74	28.40	<0.01
TID_40496	An15g02410	Has domain(s) with predicted nucleotide binding activity	3.71	2.98	4.41	1.72	0.59
TID_40740	An15g05990	Has domain(s) with predicted nucleotide binding..activity	0.25	0.48	0.23	3.69	0.60
TID_56084	An11g10890	Aldose 1-epimerase	2.37	10.48	0.50	9.75	<0.01
**Transporters**							
TID_56643	An12g09270	Putative lactose permease	0.12	38.20	1.72	676.84	<0.01
TID_38375	An08g04040	transmembrane transporter activity	0.53	7.98	0.84	32.31	<0.01
TID_55668	An06g00560	transmembrane transporter activity	0.19	24.08	0.30	263.55	<0.01
TID_180069	An07g02540	transmembrane transporter activity	0.10	2.05	0.75	43.05	<0.01
TID_197549	An02g08230	transmembrane transporter activity	0.26	18.83	0.09	146.66	<0.01
TID_54095	An18g01700	transmembrane transporter activity	0.09	2.83	0.15	65.55	<0.01
TID_54838	An13g03110	Has domain(s) with predicted role in transmembrane transport	0.35	5.77	0.11	34.61	<0.01
**Others**							
TID_120161	An18g05500	Ortholog(s) have extracellular region localization	0.64	16.86	0.14	56.34	<0.01
TID_42809	An18g03380	Ortholog(s) have IgE binding activity	0.13	4.23	1.31	69.84	<0.01
TID_180489	An07g00070	Protein of unknown function	2.84	1.66	2.29	1.21	1.00
TID_53013	An11g07040	Ortholog of A. niger ATCC 1015 : 53013-mRNA	0.07	8.61	0.10	233.95	<0.01
TID_43786	An12g02560	N/A	0.13	0.18	0.17	3.20	0.92

^a^The annotations here are for the *A. niger* CBS513.88 strain from the AspGD database [44].

^b^The fold changes were calculated by the DESeq statistical analysis package using the normalised read counts.

The surface-interacting proteins category consisted of 4 genes with two of these genes encoding hydrophobins, another gene (TID_188224) which is a homolog of a hydrophobic surface binding protein *hsbA* from *A. oryzae* [35] and one gene (TID_54125) which encoded a protein with homology to a transmembrane protein Pth11p of *Magnoporthe grisea* [36] *.* All of these four proteins have homology to proteins involved in the sensing of, or physical association with, hydrophobic surfaces and promotion of substrate degradation [35],[37]. Transcripts from three of these genes had higher expression levels in the presence of willow as compared to glucose media, with the exception being the hydrophobin-encoding TID_128530. However, the only member of this category expressed on willow at an RPKM of >10 was TID_188224 at 721 RPKM, further supporting the hypothesis that this protein may play a role in fungal response to lignocellulose. Interestingly, the expression in response to willow was 32-fold greater than that to straw (DESeq, p < 0.01).

Of the carbon and nitrogen metabolising category, only 2 of the 5 genes had transcript levels raised by >20-fold by wheat straw and willow (TID_51997 and TID_52460). This, in a similar manner to the CAZy response, could reflect differences in composition of the substrates and the small metabolites released during their degradation. All genes within the transporters category had transcript levels raised >20-fold on willow. In our previous study with straw, five other genes were induced on straw but could not be easily categorised so we categorised as ‘Others’. One of these genes TID_120161, was induced on willow with expression > 10 RPKM. The gene TID_120161 encodes a protein with homology to mitochondrial ceramidase.

The non-CAZy genes in Table 4 that are induced on willow but are not yet characterised are good candidates for future study. Overall the comparison between substrate induction profiles shown is similar for both CAZy family genes and non-CAZy related genes, insofar as the broad picture is of a similar response to willow and wheat straw by *A. niger* with differences at the level of individual genes.

## Conclusions

In the context of enzyme production for use in second generation biofuel processes, the data clearly demonstrate two key points. Firstly, the inducing substrate used for the production of enzymes has marked effects upon the enzyme cocktail achieved highlighting the limitations of the use of simple inducing substrates for the generation of complex enzyme mixtures. Secondly, the relationship between the inducing substrate and the activity of the cocktail secreted from this induction is not straightforward. For example, the wheat straw-induced cocktail was more effective at saccharifying straw than the willow-induced cocktail but the willow-induced cocktail was not more effective as saccharifying willow than the wheat straw-induced cocktail. While the cost effectiveness of using simple inducing substrates together with de-repressed fungal strains cultured on glucose-containing media has clear attractions, fungi have an enzymatic capability that can currently best be realised with induction by complex substrates. As more detailed knowledge of the complexities of how fungi efficiently saccharify lignocelluloses emerges, it will become possible to incorporate that knowledge into production procedures for more efficient and complex enzymatic cocktails. Also, the effectiveness of wheat straw as a general inducing substrate compared with other potential biofuel feedstocks could also be explored. Wheat straw induces a robust response, with good activity towards a variety of cellulose and isolated hemi-cellulosic substrates as well as towards willow and wheat straw.

## Methods

### Substrate preparation

Short rotation coppice willow (*Salix* sp.) Tora variety stems were knife-milled using a Fritsch pulverisette 19 knife-mill (Fritsch, Germany) by first passing the stems through a 2 mm screen and then a 0.5 mm screen for size reduction prior to ball-milling. 5 g of knife-milled willow was ball-milled in 80 mL stainless steel grinding bowls with 25 10-mm-diameter steel balls in a Planetary Mill (Pulverisette 5 classic line, Fritsch, Germany), at 400 rpm for a grinding time of 20 min, resulting in an average particle size of <75 μm. The wheat straw substrate preparation was described previously [5].

### Substrate compositional analysis

The total sugar in processed ball milled biomass was quantified in the hydrosylate after acid hydrolysis [38]. 30 mg of dried ball milled biomass was weighed and subjected to a two stage acid hydrolysis initially with 12 M sulphuric acid for 1 hour at 37°C followed by 1 M sulphuric acid for 2 hours at 100°C. The monosaccharide analysis was performed on fully acid hydrolysed residues and high-performance anion exchange chromatography with pulsed amperometric detection (HPAEC-PAD) (Dionex, UK) using a CarboPac PA20 column with 50 mM NaOH isocratic system at working flow rate of 0.5 ml/min at 30°C. Glucose, xylose, arabinose and galactose were used as standards with mannitol as an internal standard. The acetyl bromide method was performed to quantify lignin in ball milled biomass [39]. The details of the lignin analysis method are the same as described previously [5]. The sugar and lignin analyses were performed using three technical replicates.

### Strains and growth conditions

The *A. niger* strain used was N402 [40] and it was routinely maintained on potato dextrose agar (Oxoid). Cultures were incubated at 28°C until they had conidiated (produced asexual spores). Spores were harvested into 0.1% (v/v) Tween 20 (Sigma). Liquid batch cultures were inoculated with spores to a final concentration of 10^6^ spores ml^−1^. *A. niger* was grown in 100 ml of minimal media [all l^−1^: NaNO_3_, 6 g; KCl, 0.52 g; MgSO_4_.7H_2_O, 0.52 g; KH_2_PO_4_, 1.52 g; Na_2_B_4_O_7_.10H_2_O, 0.008 mg; CuSO_4_.5H_2_O, 0.16 mg; FePO_4_.H_2_O, 0.16 mg; MnSO_4_.4H_2_O, 0.16 mg; NaMoO_4_.2H_2_O, 0.16 mg; ZnSO_4_, 1.6 mg] with the appropriate carbon source added to a final concentration of 1% (w/v) in 250 ml Erlenmeyer flasks at 28°C, shaken at 150 rpm. The standard time-course consisted of growth from spores for 48 h in 1% (w/v) glucose media, after which mycelia were removed by filtration through Miracloth (Merck), washed thoroughly with media devoid of carbon source, and transferred to fresh media containing autoclaved 1% (w/v) ball-milled willow or wheat straw [5] as sole carbon source. Incubation was continued for 24 h. For the RNA-seq study, RNA from two further cultures was extracted where glucose (at 1% (w/v) was added to the willow cultures after 24 h and the incubation continued for a further 5 h.

### RNA extraction

Mycelia from duplicate independent cultures for each condition were frozen and ground under liquid nitrogen using a mortar and pestle, then RNA purified using the Plant/Fungi total RNA Purification Kit (Norgen Biotek, Canada) including the on-column DNase treatment step. The concentration and quality of RNA for each sample was determined by UV spectrometry (Nanodrop ND-1000 spectrophotometer).

### qRT-PCR

SuperScript™ III Reverse Transcriptase (Invitrogen) was used to synthesise cDNA from total RNA according to manufacturer’s instructions, using oligo (dT) as primer. 0.5 μg of total RNA was used for each reverse transcription. Quantitative RT-PCR amplifications were carried out using the Applied Biosystems 7500 Fast Real-Time PCR system. The PCR reaction mixture (10 μl) contained 1 μl of cDNA, specific primer sets (200 nM final concentration), and FAST SYBR-Green Master Mix (Applied Biosystems). PCRs were conducted with an initial denaturation at 95°C for 20 s, followed by 40 cycles of denaturation at 95°C for 3 s and annealing and elongation at 60°C for 30 s. Three biological replicates (shake flasks) were assayed with duplicate technical repeats of the qRT-PCR reactions. The specificity of primer sets used for qRT-PCR amplification was evaluated by melting curve analysis. The relative standard curve method was used for relative quantification using genomic DNA as the standard. All primers used, and sequences are listed in Additional file 6.

### RNA sequencing

A total of 10 μg of total RNA was depleted of ribosomal RNA using the Ribominus Eukaryotic kit (Invitrogen). Transcriptome libraries were prepared and sequenced as described [5] with the following exceptions; the KAPA library quantification kit for Life Technologies SOLiD platform was used to quantify the libraries by qPCR. Emulsion PCR and bead-based enrichment was carried out using the SOLiD EZ bead system. Using a SOLiD 5500xl ABi sequencer, 50bp/35bp paired-end reads were generated in colour space.

### Read mapping and quantification

The Life Technologies LifeScope (v2.5.1) Whole Transcriptome (WT) Pipeline was used to filter, then map the SOLiD reads to the reference genome sequence described before [41] using the mate-pair read WT pipeline. The RNA-Seq data obtained previously [5] of *A. niger* exposed to wheat straw for 24 h, as well as the data from the corresponding glucose cultures, were reanalysed using the same WT pipeline to allow data comparison. Reads from this dataset were mapped using the single fragment WT pipeline. For both data sets, reads were initially filtered against library adaptor and barcode sequences as well as *A. niger* rRNA 5.8S, 16S, 18S & 28S sequences obtained from Genbank. Reads that passed the filter were then mapped to the reference genome sequence, and to a library of exon junction sequences derived from the genome sequence using known exon coordinates. This allowed reads that spanned exon junctions (spliced reads) to be determined. Read counts per gene were determined using the program Htseq-count (http://www-huber.embl.de/users/anders/HTSeq) using uniquely aligned reads with ≥ MAPQ20. For paired read alignments only the forward read (F3) were counted. The count information was then used to calculate normalized gene expression values as RPKM [12]. Read counts were also used as the input for calculating differentially expressed (DE) genes using the R package DESeq (version 1.9) [42]. An adjusted p*-* value of ≤0.05 was the significance threshold (*p-* value adjusted for multiple testing with the Benjamini-Hochberg procedure for false discovery rate (FDR)). Where fold changes in gene expression are described in the results from our study, the fold changes are those calculated by DESeq using the normalised count data. The RNA-Seq data obtained previously for 24 h exposure to wheat straw [5] is available at the Gene Expression Omnibus (GEO) database [43] under accession number GSE33852 and the data obtained in this study for ‘willow 24 h’ and ‘willow 24 h + glucose 5 h’ along with the corresponding glucose 48 h control cultures are available under accession number GSE62284. Additional file 1 contains the expression levels for all genes.

### Gene annotation datasets used

The CAZy annotation for *A. niger* from cazy.org [15],[16] was used (last updated July 2013). The annotations for *A. niger* genes for the CBS513.88 strain were downloaded from the AspGD database [44]. A combination of sources was used to assign genes with particular activities including the enzyme commission (EC) number annotations [10] and cazy.org annotations [11],[45]. See Additional file 7 for a summary of which activities were assigned to particular genes based on the sources listed above.

### Enzyme assays with AZCL dyed substrates

The activity of enzymes in the supernatant was measured by degrading the Azurine-Cross-linked (AZCL) polysaccharides HE-cellulose and arabinoxylan (Wheat) (Megazyme, Wicklow, Ireland). 10 mg (±0.5 mg) of the AZCL dyed substrates were incubated in a total volume of 1 ml of 100 mM sodium acetate buffer pH 4.5 with un-concentrated supernatant. A volume of 10 μl of the supernatant was used for the assays with AZCL arabinoxylan and 100 μl for the assays with AZCL HE-cellulose. The reactions were incubated for 30 min at 50°C. These conditions gave a linear response between time and dye release with the above volumes. The reactions were stopped with 500 μl 2% Tris base (pH ~8). After centrifugation, the samples were diluted if needed and the absorbance was measured at 590 nm. The activity was expressed as dye released (as measured by absorbance adjusting for any dilution made to read within the absorbance range of the spectrophotometer) per volume of supernatant assayed in 30 min.

### Saccharification assays with secreted *A. niger* proteins

Six 100 ml shake flask cultures of *A. niger* with either straw or willow substrate were incubated for 24 h at 28°C. The supernatants from two of the shake flask 100 ml cultures were combined to form a replicate of secreted proteins for the saccharification experiments. The supernatants were centrifuged to pellet any solids, then filtered through 5 μm filters (surfactant-free cellulose acetate (SFCA), Sartorius) before ~120 ml of the supernatant was concentrated to 8 ml (~15 fold concentration) with Vivaspin columns (5000 MWCO, Sartorius). The concentrated supernatants were flash frozen in liquid nitrogen and stored at -80°C. The protein contents of the concentrated supernatants were estimated with the RC DC Protein Assay (Biorad) using BSA as a standard. For the saccharification assay, solutions of 1% w/v of lignocellulose substrates in AMM media were autoclaved at 117°C in the same manner as the lignocellulose substrates were prepared for the *A. niger* lignocellulose cultures. Saccharification reactions were performed in 2ml tubes in a total volume of 1.5 ml with 15 mg of washed autoclaved substrate, equal protein quantities (55 μg) of each the concentrated supernatants, 50 mM citrate buffer pH 4.8 and sodium azide at a 0.02% w/v final concentration. Reactions were incubated for 24 h at 50°C with moderate shaking. At the end of the incubation, the enzymes were inactivated by heating at 100°C for 5 min followed by centrifugation to pellet the solids. The total reducing sugars and other reducing end groups were quantified with the DNS assay [33] using glucose as a standard. Glucose was quantified with the GOPOD assay (Megazyme). Both the DNS assay and the GOPOD assays were performed in a 96-well plate format.

### Enzyme assays with pNP substrates

Concentrated culture filtrates from the same flask cultures used for the saccharification assays were assayed using 4-Nitrophenyl-β-D-cellobioside (pNP-cel), 4-Nitrophenyl-α-L-arabinofuranoside (pNP-ara), 4-Nitrophenyl-β-D-glucopyranoside (pNP-β-glu) and 4-Nitrophenyl-β-D-xylopyranoside (pNP-xyl) (all from Sigma). With assay conditions that gave a linear response between time and pNP release, the concentrated culture filtrate was assayed in a total volume of 130 μl with 2.5 mM final concentration of the substrate in 50 mM sodium acetate pH 5.0. The reactions were incubated at 37°C for 30 min with shaking, and then stopped with 130 μl of 1 M sodium carbonate before the absorbance was measured at 405 nm with a plate reader (Biotek). The enzyme activity was expressed in nmoles pNP per minute per ug protein (nmol pNP . (min. μg protein)^−1^).

### Sugar analysis of willow media before and during incubations

The methodology for the sugar analysis of the willow media before and during incubation with *A. niger* is as described previously for the incubation with wheat straw media [5].

### PAGE gel analysis

Equal amounts of protein from the concentrated culture supernatants (S/N) were analysed by SDS-PAGE. The concentrated culture S/Ns were denatured and run on a 4-20% Tris-glycine PAGE gel (Life Technologies) as described previously [5]. A pre-stained protein ladder (NEB, Cat. # P7711S) was also run on the gel. The gel was subsequently silver stained [46].

## Additional files

## Electronic supplementary material

Additional file 1: **Full list of RPKM values and** ***p-*** **values from the statistical analysis for all genes from** ***A. niger*** **for all the conditions described in this study.** The subset of CAZy genes is also presented as a separate sheet within the workbook. (XLSX 11 MB)

Additional file 2: **Expression measured by qRT-PCR for TID_43785,** ***faeA*** **and** ***axhA*** **genes.** Relative transcript levels for TID_55136 (*axhA*), TID_43785 (a CE1 family esterase) and TID_51662 (*faeA*). The expression level in *A. niger* cultured with willow is expressed relative to the expression level in one of the *A. niger* cultures with straw replicates. The expression is normalised to two reference gene *sarA* and *act*. RNA from three replicate *A. niger* cultures with each substrate were assayed here. The error bars represent standard errors. (PDF 145 KB)

Additional file 3: **SDS-PAGE gel of denatured concentrated supernatants (S/Ns) from** ***A. niger*** **cultured with either wheat straw or willow.** The 4-20% Tris-glycine SDS-PAGE gel was loaded with 0.5 μg of denatured protein as measured by the Biorad RC DC assay in lanes 2-7 and silver-stained. Lanes 2-4 contained S/N from *A. niger* cultured with straw and lanes 5-7 contained S/N from *A. niger* cultured with willow. Differences in banding pattern were observed between the S/Ns from *A. niger* cultured with either straw or willow. Lanes 2-7 have S/N from independent shake-flask cultures. (PDF 12 MB)

Additional file 4: **Time course of saccharification of willow and wheat straw using concentrated culture supernatant.** An equal amount of protein from concentrated S/Ns from *A. niger* cultured with either straw or willow was used to saccharify straw or willow substrates for different lengths of time. The reducing end groups were quantified with the DNS assay. The reducing end groups released by equal amounts of protein from the different culture S/Ns from reactions incubated for various lengths of time are expressed per mg of the lignocellulosic substrate in the saccharification assay. The results are from an assay from the S/Ns from one of the pooled duplicate cultures with either lignocellulosic substrate. The purpose of this time-course was to determine an appropriate incubation time for subsequent experiments and 24 h was chosen. (PDF 115 KB)

Additional file 5: **Free sugars in the willow media before inoculation with** ***A. niger*** **and in the willow culture supernatants incubated with** ***A. niger.*** The free sugars in the willow media and from the culture supernatants were measured using HPLC. The sugar concentration from the willow media (autoclaved) is from a single preparation of the willow media and the sugar concentrations from the culture supernatants (3 h *A. niger* to 24 h *A. niger*) are from triplicate shake flask cultures. Error bars represent standard errors. (PDF 376 KB)

Additional file 6: **Table with primer sequences used in this study.** (PDF 676 KB)

Additional file 7: **Table with list of which genes were assigned to particular enzyme activities for use in Figures** 1 and 2. (XLSX 12 KB)

Below are the links to the authors’ original submitted files for images.

Authors’ original file for figure 1

Authors’ original file for figure 2

Authors’ original file for figure 3

Authors’ original file for figure 4

## Abbreviations

qRT-PCR: Quantitative reverse transcriptase/real-time PCR
*p*: *p-* value from DESeq analysis adjusted for multiple hypothesis testing
S/N: Supernatant
MWCO: Molecular weight cut-off
