# Supplementary figures and images for: RNA-sequencing reveals the complexities of the transcriptional response to lignocellulosic biofuel substrates in Aspergillus niger
Source: Fungal Biol Biotechnol. 2014 Nov 17;1:3. doi: 10.1186/s40694-014-0003-x (PMC5598271; doi:10.1186/s40694-014-0003-x)

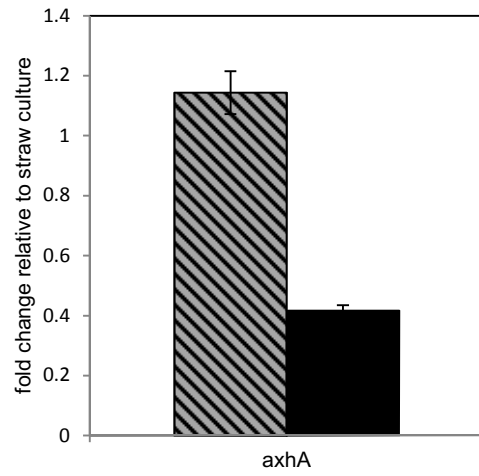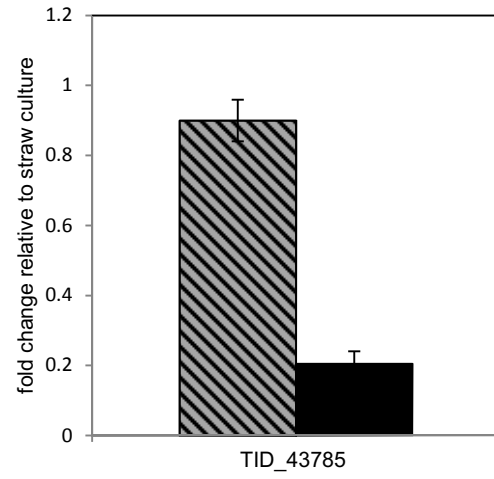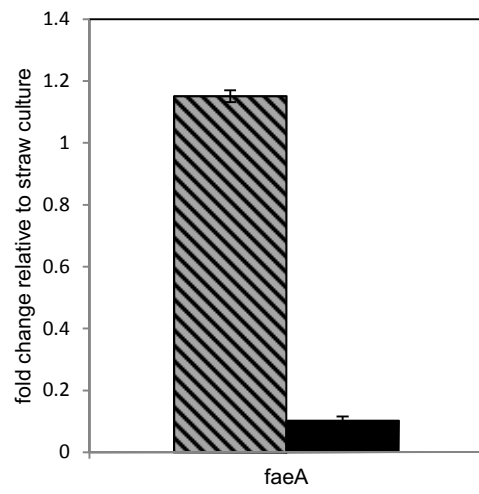

■ straw cultures  
■ willow cultures

Supplement: Supplementary file 2 — Additional file 2: Expression measured by qRT-PCR for TID_43785, faeA and axhA genes. Relative transcript levels for TID_55136 (axhA), TID_43785 (a CE1 family esterase) and TID_51662 (faeA). The expression level in A. niger cultured with willow is expressed relative to the expression level in one of the A. niger cultures with straw replicates. The expression is normalised to two reference gene sarA and act. RNA from three replicate A. niger cultures with each substrate were assayed here. The error bars represent standard errors. (PDF 145 KB) [file 40694_2014_3_MOESM2_ESM.pdf]

Lane 1 2 3 4 5 6 7

KDa

230

80

60

30

25

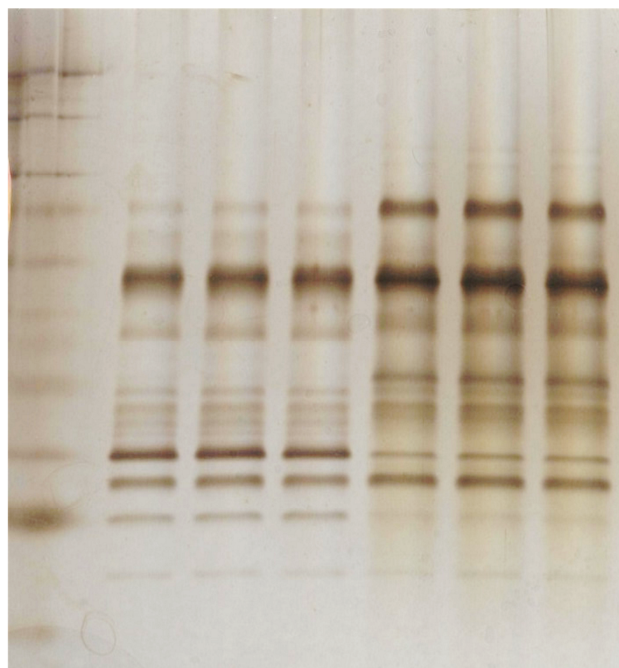

Supplement: Supplementary file 3 — Additional file 3: SDS-PAGE gel of denatured concentrated supernatants (S/Ns) from A. niger cultured with either wheat straw or willow. The 4-20% Tris-glycine SDS-PAGE gel was loaded with 0.5 μg of denatured protein as measured by the Biorad RC DC assay in lanes 2-7 and silver-stained. Lanes 2-4 contained S/N from A. niger cultured with straw and lanes 5-7 contained S/N from A. niger cultured with willow. Differences in banding pattern were observed between the S/Ns from A. niger cultured with either straw or willow. Lanes 2-7 have S/N from independent shake-flask cultures. (PDF 12 MB) [file 40694_2014_3_MOESM3_ESM.pdf]

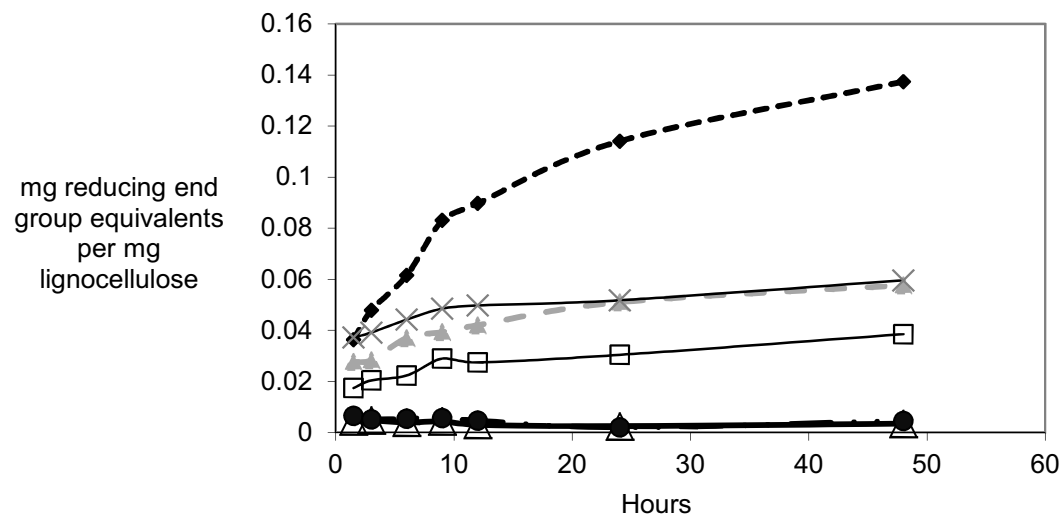

Supplement: Supplementary file 4 — Additional file 4: Time course of saccharification of willow and wheat straw using concentrated culture supernatant. An equal amount of protein from concentrated S/Ns from A. niger cultured with either straw or willow was used to saccharify straw or willow substrates for different lengths of time. The reducing end groups were quantified with the DNS assay. The reducing end groups released by equal amounts of protein from the different culture S/Ns from reactions incubated for various lengths of time are expressed per mg of the lignocellulosic substrate in the saccharification assay. The results are from an assay from the S/Ns from one of the pooled duplicate cultures with either lignocellulosic substrate. The purpose of this time-course was to determine an appropriate incubation time for subsequent experiments and 24 h was chosen. (PDF 115 KB) [file 40694_2014_3_MOESM4_ESM.pdf]

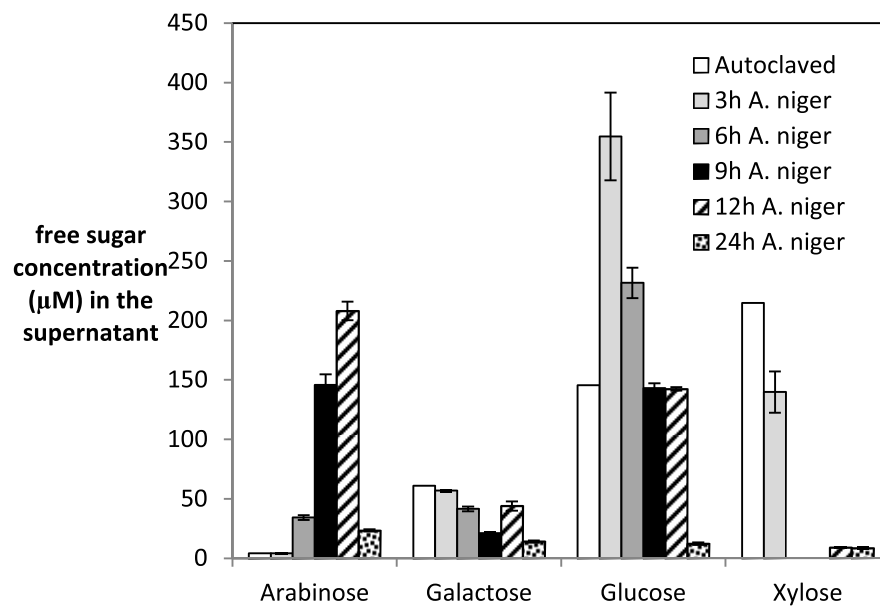

Supplement: Supplementary file 5 — Additional file 5: Free sugars in the willow media before inoculation with A. niger and in the willow culture supernatants incubated with A. niger. The free sugars in the willow media and from the culture supernatants were measured using HPLC. The sugar concentration from the willow media (autoclaved) is from a single preparation of the willow media and the sugar concentrations from the culture supernatants (3 h A. niger to 24 h A. niger) are from triplicate shake flask cultures. Error bars represent standard errors. (PDF 376 KB) [file 40694_2014_3_MOESM5_ESM.pdf]

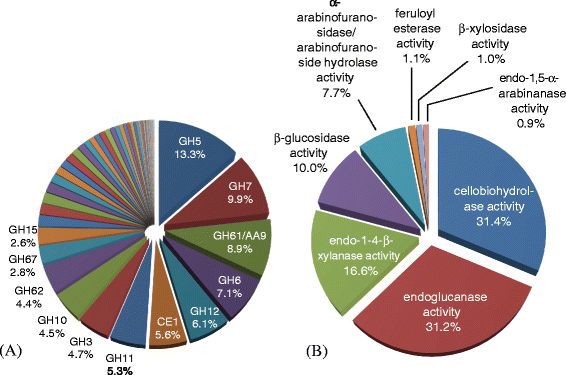

Supplement: Supplementary file 8 — Authors’ original file for figure 1 [file 40694_2014_3_MOESM8_ESM.gif]

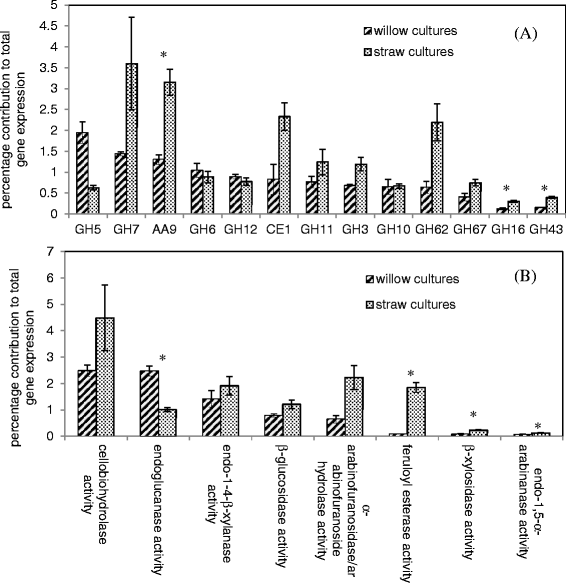

Supplement: Supplementary file 9 — Authors’ original file for figure 2 [file 40694_2014_3_MOESM9_ESM.gif]

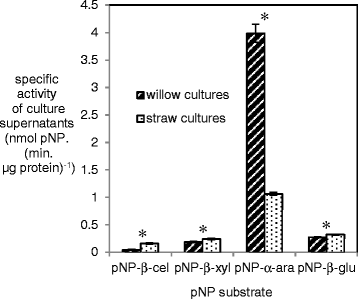

Supplement: Supplementary file 10 — Authors’ original file for figure 3 [file 40694_2014_3_MOESM10_ESM.gif]

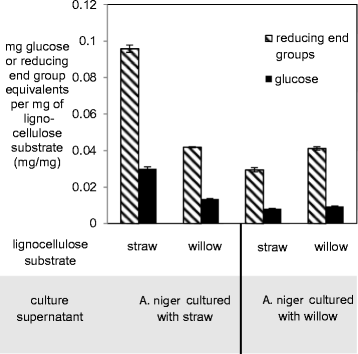

Supplement: Supplementary file 11 — Authors’ original file for figure 4 [file 40694_2014_3_MOESM11_ESM.gif]
