# Supplementary material for: RNA-sequencing reveals the complexities of the transcriptional response to lignocellulosic biofuel substrates in Aspergillus niger
Source: Fungal Biol Biotechnol. 2014 Nov 17;1:3. doi: 10.1186/s40694-014-0003-x (PMC5598271; doi:10.1186/s40694-014-0003-x)
Supplement: Supplementary file 6 — Additional file 6: Table with primer sequences used in this study. (PDF 676 KB) [file 40694_2014_3_MOESM6_ESM.pdf]

Gene name	ATCC1015 identifier	Primer name	Sequence (5'-3')
faeA	TID_51662	An09g00120_Fw	GCCCAGCTGTCCGCGACATA
		An09g00120_Rv	ATGGGCGTAACCCTGCTCCG
axhA	TID_55136	An03g00960_Fw	CGATGCTCTCGCCACCCCAA
		An03g00960_Rv	AGCGGATGCCATGTCCGACC
TID_43785	TID_43785	An12g02550_Fw	AATCTCGGGCGCAAGGCAGT
		An12g02550_Rv	AAACCCGTCGCGAAGCGAGA
act	TID_200483	An15g00560Fw	TCCTGGGTCTGGAGAGCGGTG
		An15g00560Rv	CTGCATACGGTCGGAGATACCGGG
sarA	TID_206151	An01g04040Fw	GAGGATGAGCTGCGCCACCA
		An01g04040Rv	GACAGCCATCTGATACCTTCACCGT
